# Molecular Assay Versus Serology for Diagnosis of Scrub Typhus Among Patients With Acute Febrile Illness: A Retrospective Study

**DOI:** 10.7759/cureus.76104

**Published:** 2024-12-20

**Authors:** Kumudini Panigrahi, Adrita Das, Jyoti Prakash Sahoo, Basanti Kumari Pathi, Dipti Pattnaik

**Affiliations:** 1 Microbiology, Kalinga Institute of Medical Sciences, Bhubaneswar, IND; 2 Pharmacology, Kalinga Institute of Medical Sciences, Bhubaneswar, IND

**Keywords:** correlation analysis, critical threshold value, diagnostic method, eschar, fever, heatmap, igm elisa, optical density value, real time-pcr, scrub typhus

## Abstract

Background and objectives: The epidemiology of scrub typhus caused by *Orientia tsutsugamushi* has been growing in Odisha, India. The most common symptoms include fever, cough, lymphadenopathy, eschar, and rash. In India, enzyme-linked immunosorbent assays (ELISA) and DNA real-time polymerase chain reaction (DNA RT-PCR) are the most commonly used methods to diagnose scrub typhus. We conducted this study to determine the pattern of symptoms and association between the findings of ELISA and DNA RT-PCR.

Methods: This retrospective study was accomplished at the Kalinga Institute of Medical Sciences (KIMS), Bhubaneswar, OR, IND. We analyzed the data of adult patients admitted with scrub typhus between March 2022 and February 2024, and their blood samples were evaluated with both IgM ELISA and DNA RT-PCR. The IgM antibody levels against *Orientia tsutsugamushi* were detected by ELISA and expressed as optical density (OD) values. The bacterial load was assessed by RT-PCR and presented as cycle threshold (CT) values. The OD values > 0.5 and CT values < 40 were deemed positive diagnoses for scrub typhus. The symptoms were weighed for their incidence and co-occurrence. We correlated the OD and CT values of the study population. The R software version 4.4.1 (R Foundation for Statistical Computing, Vienna, AUT) was utilized for data analysis.

Results: Of the 231 patients scrutinized, 170 were deemed eligible. Sixty-five (38.2%) participants were female. The median age of the study subjects was 44.5 (24.0-61.8) years. All of them had fever. Other symptoms (in decreasing order of incidence) were as follows: cough (139, 81.8%), lymphadenopathy (29, 17.1%), rash (26, 15.3%), abdomen pain (18, 10.6%), eschar (6, 3.5%), and seizure (2, 1.2%). Maximum co-occurrence was observed between cough, lymphadenopathy, rash, and pain in the abdomen with fever. It held good for both females and males. The study population's median OD and CT values were 2.8 (0.8-3.7) and 41.0 (36.0-42.0), respectively. The OD and CT values were positively correlated (r = 0.78, 95% CI = 0.71-0.83, p < 0.001).

Conclusion: The study participants had symptoms like fever, cough, lymphadenopathy, rash, pain in the abdomen, and eschar. Our findings suggest that ELISA and DNA RT-PCR could be used for scrub typhus diagnosis among patients with febrile illness for > 5 and ≤ 5 days, respectively. Both diagnostic methods were found to possess a positive correlation. The findings of ELISA and DNA RT-PCR must be probed further.

## Introduction

Scrub typhus, transmitted through the bacterium *Orientia tsutsugamushi*, constitutes one of the most common causes of acute fever [1,2]. It is a rickettsial infection transmitted through the bite of mites, e.g., Leptotrombidium deliense and Leptotrombidium akamushi (also known as Trombicula akamushi) [3]. These mites are typically found in scrub vegetation environments [1-3]. The term "scrub" in scrub typhus refers to the vegetation that nurtures the chigger-host link [2]. Wild rats and other small rodents infect the mite in its larval stage (also known as chigger). Through the monsoon season, scrub typhus gets introduced to humans via the bite of an infected chigger. Scrub typhus frequently resembles dengue and malaria [2,3]. Scrub typhus has recently spread throughout India [4,5]. In 2023, the Indian states of Himachal Pradesh (with nine fatalities) and Odisha (with eight fatalities) reported significantly higher levels of scrub typhus cases [3]. The scrub typhus incubation period spans six to 21 days [6,7]. Farmers and army soldiers are among the populations at risk [3,4].

Acute fever, headache, lymphadenopathy, coughing, rash, seizures, and upset stomach are typical manifestations [3,8,9]. Patients might have an eschar as well. Eschar is a tiny, necrotic lesion with a dark center that could look like a burn mark from cigarettes [8,9]. Frequently, these are located on the neck, genitalia, axilla, or groin. Despite being pathognomonic for scrub typhus, the prevalence of eschar varies significantly, ranging from 7% to 97% [10]. Due to its non-extensive epidemiology among Indians and other Asian people, eschar is not regarded as a suitable approach to diagnose scrub typhus [11]. Therefore, laboratory tests are performed to confirm the diagnosis.

Serological tests such as the Weil-Felix test, indirect immunoperoxidase assays, indirect immunofluorescence assays, enzyme-linked immunosorbent assays (ELISA), and immunochromatographic tests (ICT) are used in the laboratory to diagnose scrub typhus. Nowadays, ELISA is more preferred and widely performed than other serological tests [12-14]. Direct diagnostic techniques include bacterial culture in chick embryo cell lines and molecular assays like real-time polymerase chain reaction (RT-PCR). The molecular assay, i.e., the DNA real-time polymerase chain reaction (DNA RT-PCR) method, targets several genetic markers, including the *16s rRNA*, *47-kDa high-temperature requirement A (HtrA)*, *56-kDa *type-specific antigen (TSA), and *GroEL* genes [15]. Direct diagnostic approaches are superior to indirect ones due to their higher sensitivity and specificity in identifying infections in the early stages. *Orientia tsutsugamushi *culture mandates biosafety level (BSL) 3 facilities [16]. Nowadays, ELISA and DNA RT-PCR are India's two most commonly leveraged methods for diagnosing scrub typhus. The choice between ELISA and DNA RT-PCR relies on resource availability [17,18]. We conducted this study to determine the symptom patterns of patients with scrub typhus and the correlation between their findings of ELISA and DNA RT-PCR tests.

## Materials and methods

In this retrospective study, we investigated the case records of patients with scrub typhus for their symptoms and findings of ELISA and DNA RT-PCR tests. The study was conducted at Kalinga Institute of Medical Sciences (KIMS), Bhubaneswar, OR, IND, from March 2022 until February 2024. We had ethical permission from the Institutional Ethics Committee before initiating the study (approval no. KIIT/KIMS/IEC/1755/2024 dated 25.03.2024). The research adhered to institutional standards, good clinical practices, good laboratory practices, and the Declaration of Helsinki.

Study participants

In this study, we included adult patients with scrub typhus admitted to our hospital during the stipulated period and evaluated with both IgM ELISA and DNA RT-PCR. The blood samples were regarded as positive when the optical density (OD) value of IgM ELISA was > 0.5 or the cycle threshold (CT) value of DNA RT-PCR was < 40. The patients were deemed eligible if their scrub typhus diagnosis was aided by either IgM ELISA, DNA RT-PCR, or both. Patients with blood reports negative for scrub typhus or positive for other acute febrile illnesses such as malaria, dengue fever, chikungunya fever, enteric fever, and urinary tract infection (UTI) were excluded from this study.

Study procedure

The demographic (e.g., age, gender), clinical (e.g., number and duration of symptoms) traits, and laboratory parameters (e.g., scrub typhus IgM ELISA and DNA RT-PCR findings) of eligible participants were recorded. The socioeconomic status of the participants was computed with the Kuppuswamy classification. A pair of whole blood samples from the suspected patients were collected and sent to the microbiology laboratory for serological and molecular testing (scrub typhus IgM ELISA and DNA RT-PCR), maintaining a cold chain. The diagnostic tests were performed per the manufacturer's instructions.

IgM ELISA for *O. tsutsugamushi*

The commercial kit Scrub Typhus Detect™ IgM ELISA (InBios International Inc., Seattle, WA, USA) was used. This kit uses recombinant 56-kDa TSA of *O. tsutsugamushi* Karp, Kato, Gilliam, and TA716 strains to detect scrub typhus IgM antibodies. Before commencing the process, the kit's reagents were brought to room temperature. The kit's sample dilution buffer was deployed to dilute the serum samples at 1:100. Positive controls and diluted serum were poured into the antigen-coated microwells. The Bio-Rad® (Bio-Rad Laboratories, Hercules, CA, USA) ELISA reader (PR 4100) was used to measure the OD values of the microwells in the plate at 450 nm. This method detects IgM antibody levels against *O. tsutsugamushi*. The antibody titer rises after five days of onset of scrub typhus. Hence, a higher IgM antibody titer indicates a recent scrub typhus infection. A duplicate reading was obtained for every well. Scrub typhus had a computed cutoff value of 0.5. The OD values > 0.5 were regarded as reactive. Those with OD values < 0.5 were deemed non-reactive. For equivocal reports, the assays were repeated. The patients with fever for > 5 days were expected to have higher OD values than those with fever for ≤ 5 days.

RT-PCR test for DNA of *O. tsutsugamushi*

The targeted genes in the blood samples to detect *O. tsutsugamushi* were *16s rRNA* and *47-kDa HtrA* genes. The DNA extraction was done using the TRUPCR® (TRUPCR Europe, Manchester, GBR) blood DNA extraction kit. The extracted DNA samples were stored at -20°C. For PCR reaction mix preparation, TRUPCR® master mix and primer probes were mixed for the required number of tests. Fifteen µl of the PCR mixture was put into the PCR tubes of 0.2 ml volume. Ten µl of extracted DNA (from the clinical sample) was added to each PCR tube. The positive and negative controls provided in the kit were added to the PCR tubes for a total volume of 25 µl.

The Applied Biosystem QuantStudio™ 5 (QS-5) (Thermo Fisher Scientific, Waltham, MA, USA) real-time PCR system was used. The prepared samples were run through this system. The assay runs for a maximum of 45 cycles. For the RT-PCR cycler QS-5, a CT value of 40 was established. The internal control had a threshold cycle value of 32, which validated our system. The negative controls without DNA did not impart any fluorescence. The bacteria are expected to provide a CT value to prove the magnitude of their presence. A higher bacterial load yields a lower CT value than a lower one. This bacterial load starts to fall five days after the onset of scrub typhus, along with the rising antibody titer. Hence, receding bacterial load or increasing CT value indicates a recent scrub typhus infection. The patients with fever for > 5 days were expected to have higher CT values than those with fever for ≤ 5 days.

Fluorescence signals were recorded through a fluorometric thermocycler: the test sample and internal control imparted fluorescence. The internal control channels were identified as ROX/red, and *O. tsutsugamushi* DNA channels as FAM/green. The test sample was deemed positive for *O. tsutsugamushi* DNA if the test sample and positive control had positive amplification. The test sample was regarded as negative for *O. tsutsugamushi *DNA if the test sample had negative and positive control had positive amplification. If the test sample and positive control had negative amplification, the assay was performed again.

Statistical analysis

We used convenience sampling for this retrospective study. The Shapiro-Wilk test was deployed to confirm the normality of the data distribution. The continuous data were expressed with median and interquartile range (IQR). The frequency and proportion were displayed for the categorical data. We displayed the clinical symptoms through scatter plots. The quantitative variables, such as age, OD, and CT values, were illustrated via box-whisker plots. To demonstrate the co-occurrence of symptoms, we leveraged a heatmap diagram. We used Pearson's correlation to evaluate the association between OD and CT values. We also performed a subgroup analysis of the quantitative variables based on the fever duration. Version 4.4.1 of the R software (R Foundation for Statistical Computing, Vienna, AUT) was utilized for data computation [19]. The p values < 0.05 were regarded as statistically significant.

## Results

A total of 231 patients were admitted to our hospital with a diagnosis of scrub typhus during the study period (i.e., March 2022 to February 2024). Of them, 23 (9.96%) had UTIs, 18 (7.79%) subjects were diagnosed with dengue, 13 (5.63%) patients had an additional diagnosis of enteric fever, and three (1.30%) and two (0.87%) patients were additionally diagnosed with malaria and chikungunya fever, respectively. The remaining 170 scrub typhus patients diagnosed with either IgM ELISA, DNA RT-PCR, or both were analyzed in this retrospective study. Table 1 displays the sociodemographic and clinical characteristics of the study participants. Of the participants, 65 (38.2%) were women. The study participants' median age was 44.5 (24.0-61.8) years. Of the participants, 98 (57.6%) belonged to lower socioeconomic classes, 61 (35.9%) were from the lower-middle class, and 11 (6.5%) were from the upper-middle class. A total of 73 (42.9%) and 47 (27.6%) participants had type 2 diabetes mellitus and hypertension, respectively. All study participants had a raised temperature > 101°F during admission. Other symptoms encountered among the study participants were cough (139, 81.8%), lymphadenopathy (29, 17.1%), rash (26, 15.3%), abdominal pain (18, 10.6%), eschar (six, 3.5%), and seizure (two, 1.2%). The study population's median CT and OD values were 41.0 (36.0-42.0) and 2.8 (0.8-3.7), respectively.

**Table 1:** Sociodemographic and clinical characteristics of the study population The median (IQR) and frequency (proportion) were used to represent the continuous and categorical data, respectively. IQR: interquartile range, OD: Optical density, ELISA: Enzyme-linked immunosorbent assay, CT: Cycle threshold, RT-PCR: Real-time polymerase chain reaction

Parameter	Value
Total participants	170
Age (years)	44.5 (24.0-61.8)
Female	65 (38.2%)
Socioeconomic class	
Lower	98 (57.6%)
Lower-middle	61 (35.9%)
Upper-middle	11 (6.5%)
Comorbidities	
Type 2 diabetes mellitus	73 (42.9%)
Hypertension	47 (27.6%)
Symptoms	
Fever	170 (100%)
Fever ≤ 5 days	49 (28.8%)
Fever > 5 days	96 (71.2%)
Cough	139 (81.8%)
Lymphadenopathy	29 (17.1%)
Rash	26 (15.3%)
Pain abdomen	18 (10.6%)
Eschar	6 (3.5%)
Seizure	2 (1.2%)
OD value (assessed with ELISA)	2.8 (0.8-3.7)
CT value (assessed with DNA RT-PCR)	41.0 (36.0-42.0)

**Figure 1:**
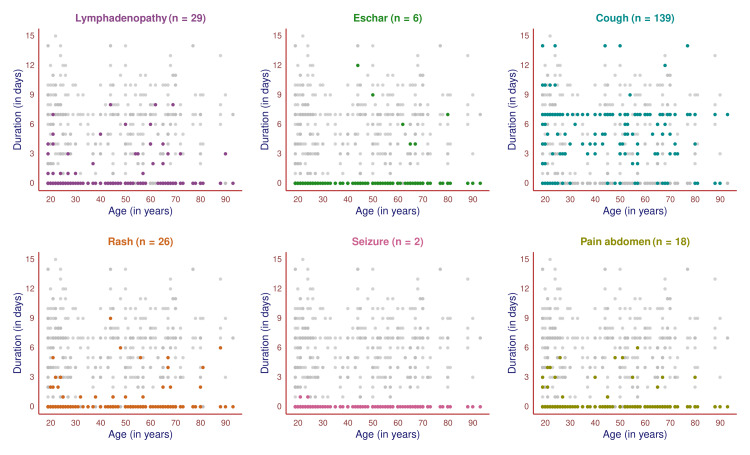
Symptoms of scrub typhus among the study participants (n = 170) The scatter plots portray the pattern of all symptoms (except fever) of the study participants in different colors. The x-axis and y-axis denote the individual's age (in years) and symptom duration (in days).

The scatter plot in Figure 1 showcases the symptoms of 170 study participants and their durations. All participants had febrile illness. The remaining six symptoms were highlighted in different colors to illustrate the number of participants experiencing the symptoms, the age of the participants, and the duration of the symptoms for each participant. The most common symptom after fever was cough (139, 81.8%), followed by lymphadenopathy (29, 17.1%), rash (26, 15.3%), pain in the abdomen (18, 10.6%), eschar (six, 3.5%), and seizure (two, 1.2%). The cough had more chronicity than any other symptoms. Eschar affected only the elder subjects. In this study, two younger participants had single seizure episodes one day before being hospitalized.

**Figure 2:**
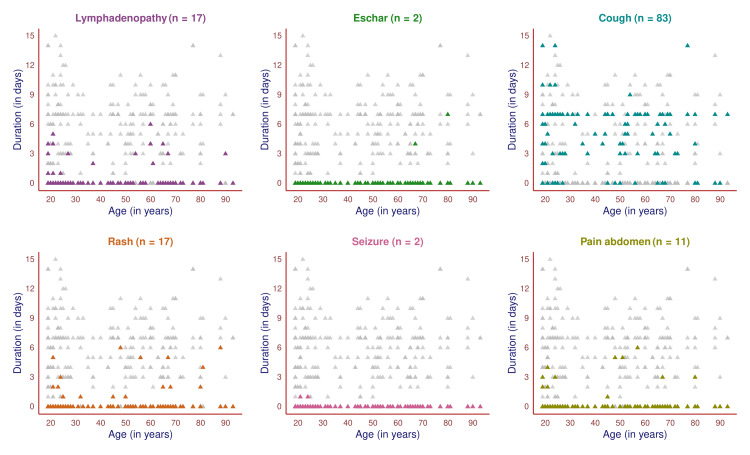
Symptoms of scrub typhus among male participants (n = 105) The scatter plots portray the pattern of all male subjects' symptoms (except fever) in different colors. The x-axis and y-axis denote the participant's age (in years) and symptom duration (in days).

The scatter plot in Figure 2 showcases the symptoms of 105 male participants and their durations. All of them had febrile illness. The remaining six symptoms were highlighted in different colors to illustrate the number of male participants experiencing the symptom, their age, and the duration of symptoms. The most common symptom after the fever was cough (83, 79.0%), followed by lymphadenopathy (17, 16.2%), rash (17, 16.2%), pain in the abdomen (11, 10.5%), eschar (two, 1.9%), and seizure (two, 1.9%). The cough had more chronicity than any other symptoms. Eschar and seizures were noticed in two elderly and two younger males. None of the 105 male participants experienced any symptoms (except cough) for over a week.

**Figure 3:**
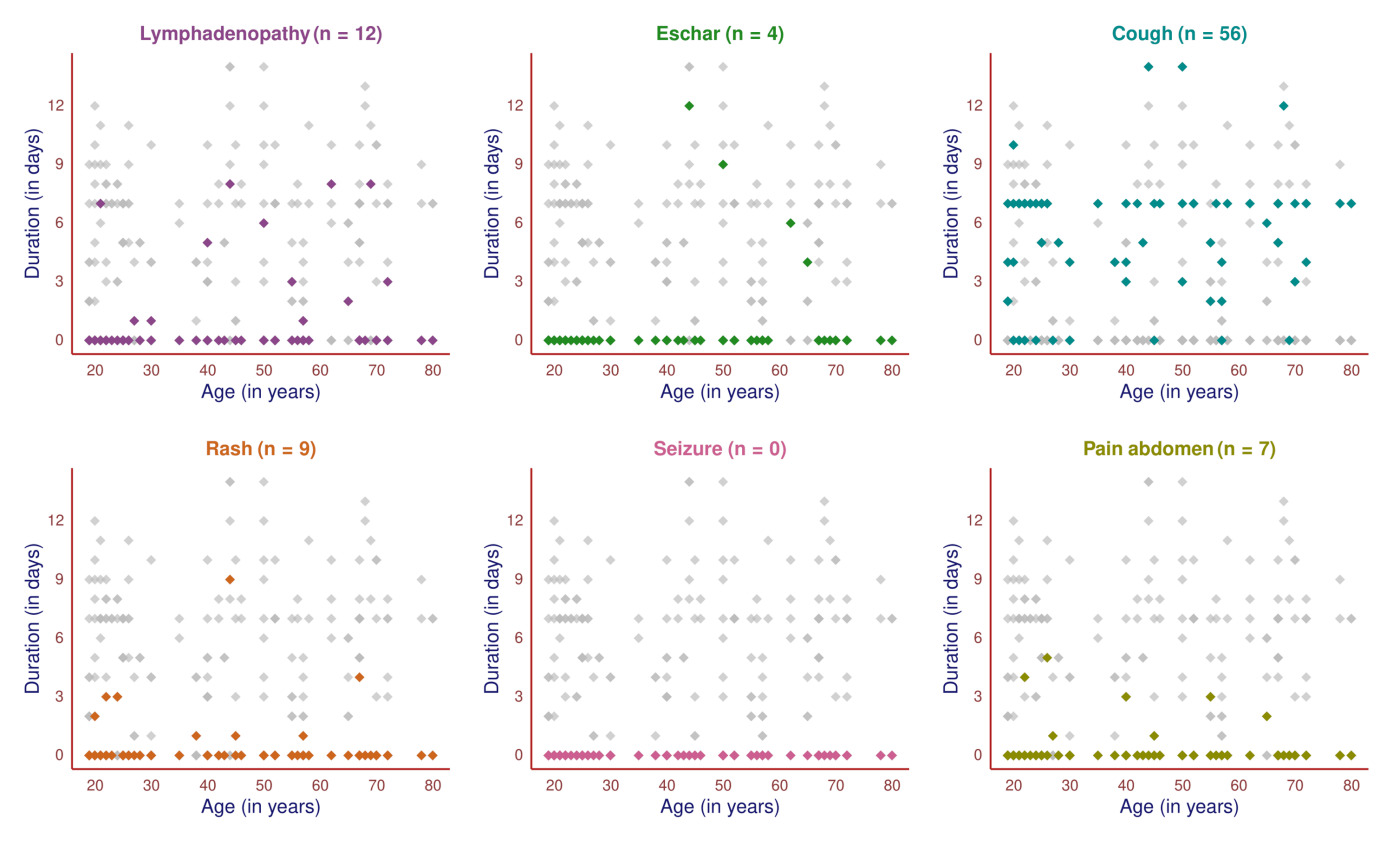
Symptoms of scrub typhus among female participants (n = 65) The scatter plots portray the pattern of all symptoms (except fever) of female participants in different colors. The x-axis and y-axis denote the subject's age (in years) and symptom duration (in days).

The scatter plot in Figure 3 showcases the symptoms of 65 female participants and their durations. All of them had febrile illness. The remaining six symptoms are highlighted in different colors to illustrate the number of female participants experiencing the symptom, their age, and the duration of symptoms. The most common symptom after the fever was cough (56, 86.2%), followed by lymphadenopathy (12, 18.5%), rash (nine, 13.8%), pain in the abdomen (seven, 10.8%), and eschar (four, 6.2%). The cough had more chronicity than any other symptoms. None of the female participants had seizures. The symptoms were more chronic in females when juxtaposed with male subjects.

**Figure 4:**
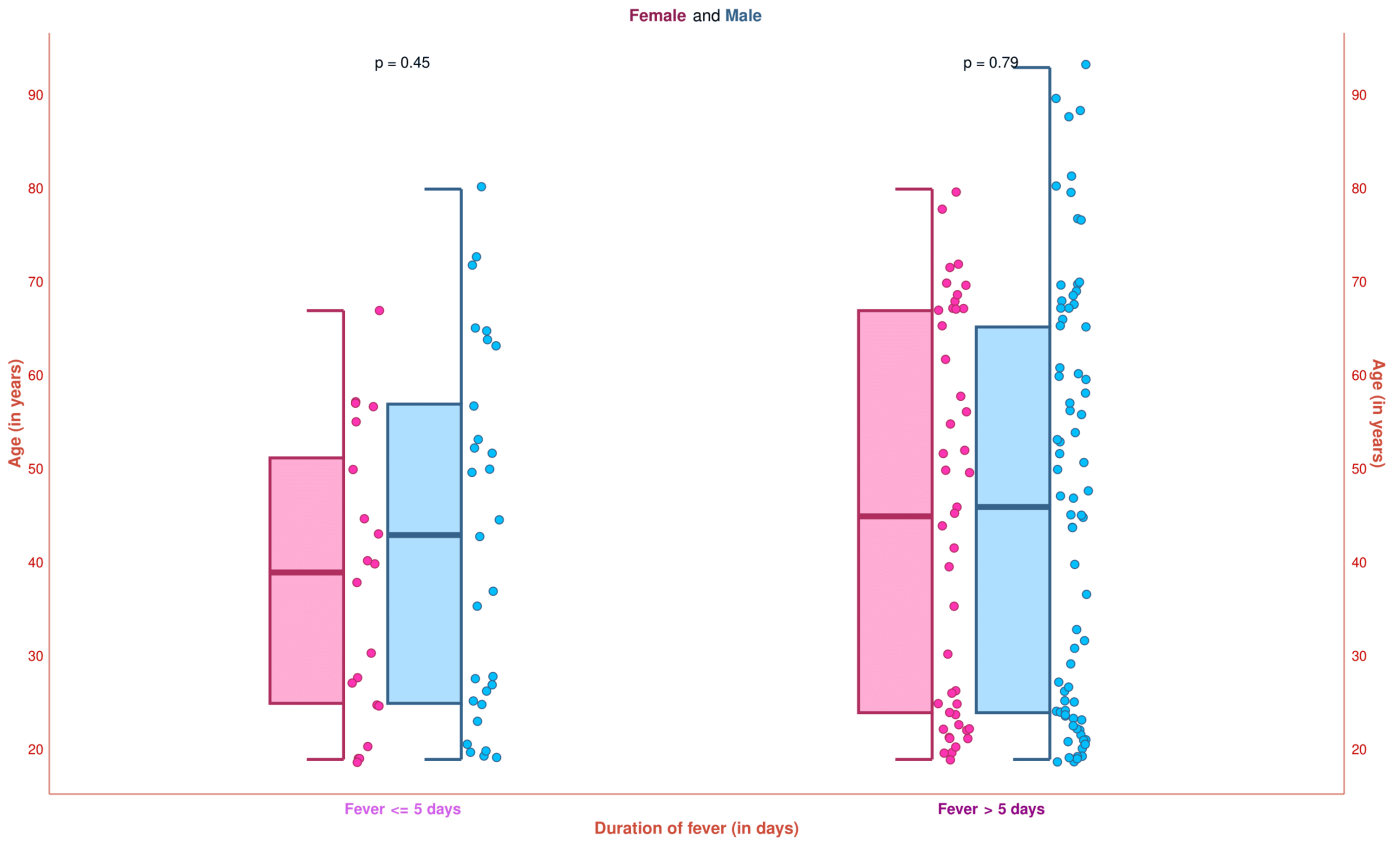
Age distribution of the study population The half-box-whisker and jitter plots demonstrate the age distribution of the study participants. The x-axis and y-axis denote the participants’ duration of fever (in days) and age (in years), respectively.

The half-box-whisker and jitter plots in Figure 4 illustrate the age distribution of the study participants. The median age of the study population was 44.5 (24.0-61.8) years (female: 42.0 (24.0-57.0) years; male: 45.0 (24.0-64.0) years). Subgroup analysis was performed based on fever duration. The median ages for the 20 female and 29 male participants with fever ≤ 5 days were 39.0 (25.0-51.3) years and 43.0 (25.0-57.0) years, respectively (p = 0.45). The median ages for the 45 female and 76 male participants having fever > 5 days were 45.0 (24.0-67.0) years and 46.0 (24.0-65.0) years, respectively (p = 0.79). These findings indicate that most of the study population had febrile illness for over five days. The incidence of chronic fever was higher in elderly individuals.

**Figure 5:**
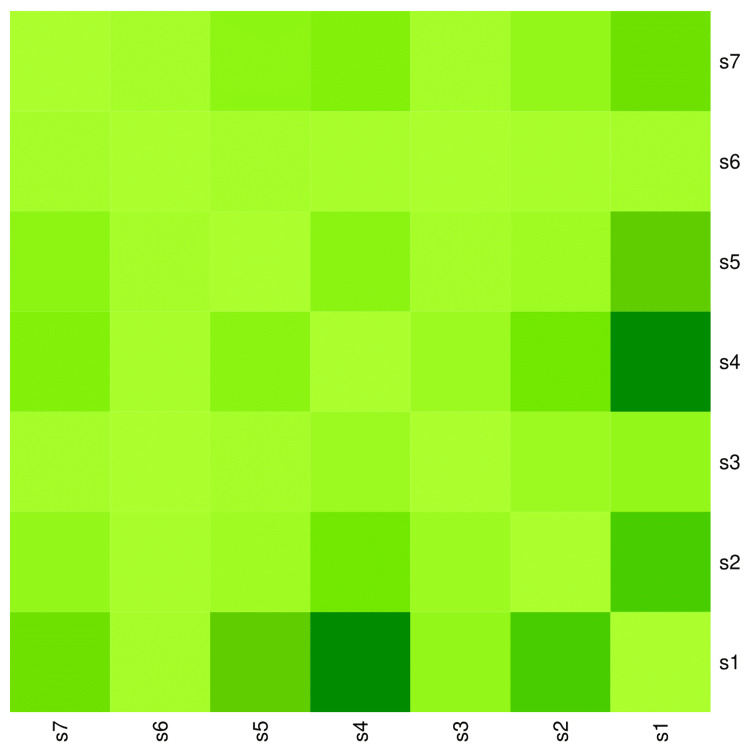
Co-occurrence of symptoms among the study participants The heatmap diagram illustrates the co-occurrence of various symptoms of scrub typhus among the study participants. Both the x-axis and y-axis represent the seven symptoms observed among the study participants. The bottom-left and top-right portions of the diagram are diagonally symmetrical. The lighter shade of green indicates the degree of co-occurrence of symptoms. Darker shades of green denote a higher degree of co-occurrence of symptoms. s1: Fever, s2: Lymphadenopathy, s3: Eschar, s4: Cough, s5: Rash, s6: Seizure, s7: Abdomen pain

The heatmap diagram in Figure 5 showcases the co-occurrence of various symptoms among the 170 participants. The bottom-left and top-right segments of the heatmap are diagonally symmetrical. The highest co-occurrence was observed between fever and cough (139, 81.8%), followed by fever and lymphadenopathy (29, 17.1%), fever and rash (26, 15.3%), fever and pain abdomen (18, 10.6%), lymphadenopathy and cough (15, 8.8%), and cough and abdomen pain (10, 5.9%).

**Figure 6:**
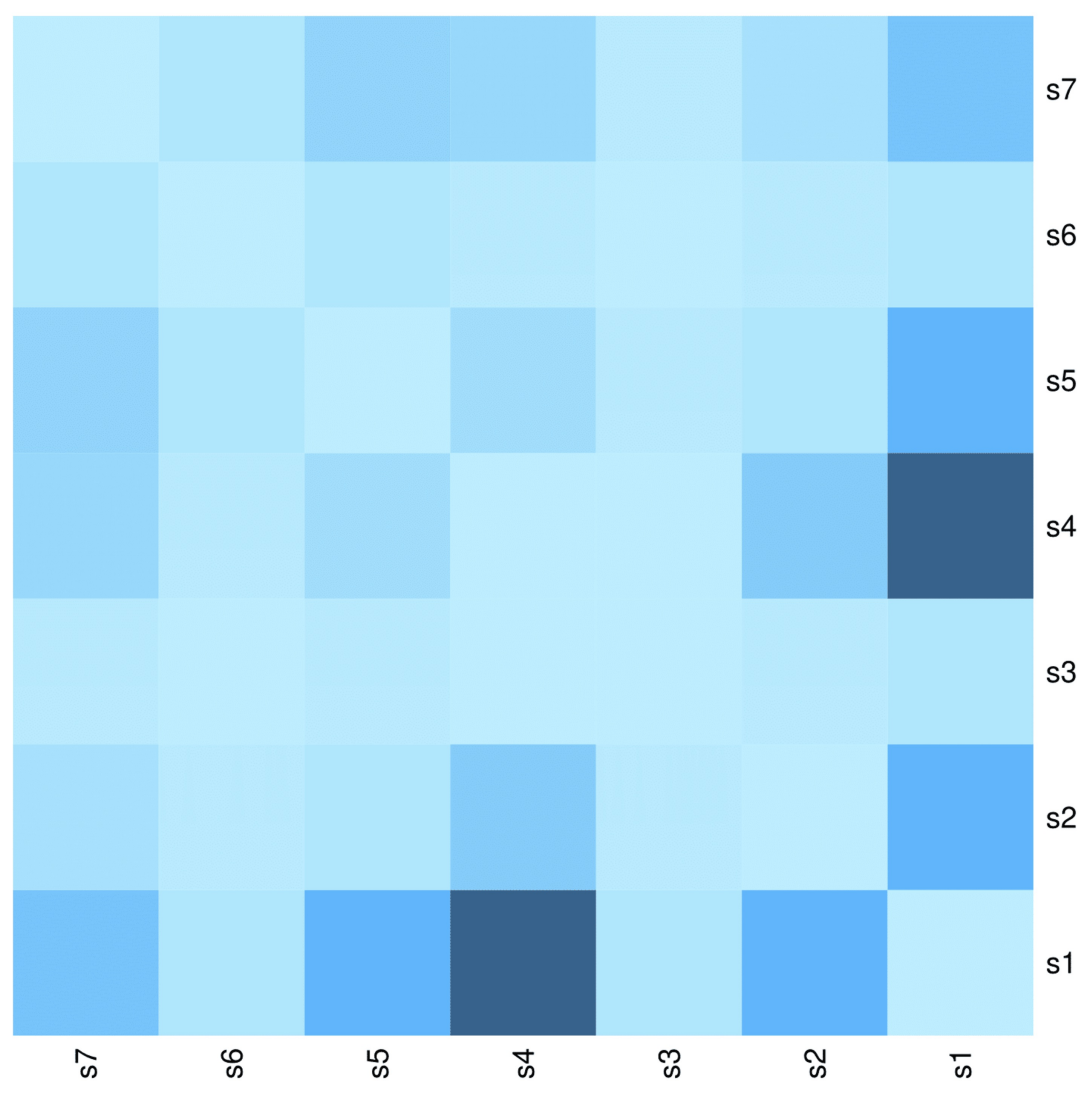
Co-occurrence of symptoms among male participants The heatmap diagram illustrates the co-occurrence of various symptoms of scrub typhus among the 105 male participants. Both the x-axis and y-axis represent the seven symptoms observed among male participants. The bottom-left and top-right portions of the diagram are diagonally symmetrical. The lightest shade of blue indicates the degree of co-occurrence of symptoms. Darker shades of blue denote a higher degree of co-occurrence of symptoms. s1: Fever, s2: Lymphadenopathy, s3: Eschar, s4: Cough, s5: Rash, s6: Seizure, s7: Abdomen pain

The heatmap diagram in Figure 6 showcases the co-occurrence of various symptoms among the 105 male participants. The bottom-left and top-right segments of the heatmap are diagonally symmetrical. Among the male participants, the highest co-occurrence was observed between fever and cough (83, 79.0%), followed by fever and lymphadenopathy (17, 16.2%), fever and rash (17, 16.2%), fever and abdomen pain (11, 10.5%), lymphadenopathy and cough (eight, 7.6%), and rash and abdomen pain (six, 5.7%). The co-occurrence pattern of the symptoms matches that of the entire study population.

**Figure 7:**
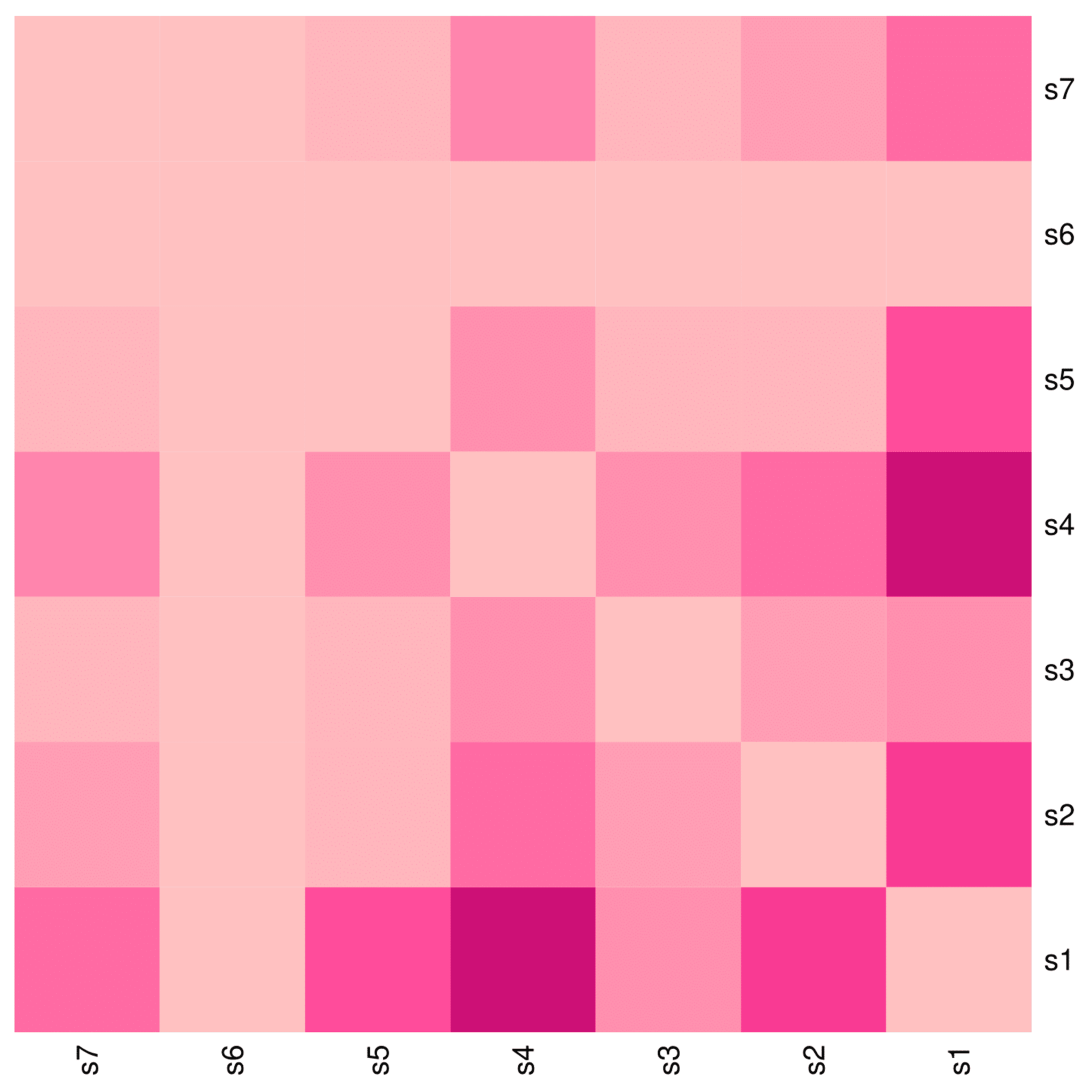
Co-occurrence of symptoms among female participants The heatmap diagram illustrates the co-occurrence of various symptoms of scrub typhus among the 65 female participants. Both the x-axis and y-axis represent the seven symptoms observed among female participants. The bottom-left and top-right portions of the diagram are diagonally symmetrical. The lighter shade of pink indicates the degree of co-occurrence of symptoms. Darker shades of pink denote a higher degree of co-occurrence of symptoms. s1: Fever, s2: Lymphadenopathy, s3: Eschar, s4: Cough, s5: Rash, s6: Seizure, s7: Abdomen pain

The heatmap diagram in Figure 7 showcases the co-occurrence of various symptoms among the 65 female participants. The bottom-left and top-right segments of the heatmap are diagonally symmetrical. Among the female participants, the highest co-occurrence was observed between fever and cough (56, 86.2%), followed by fever and lymphadenopathy (12, 18.5%), fever and rash (nine, 13.8%), fever and abdomen pain (seven, 10.8%), lymphadenopathy and cough (seven, 10.8%), and cough and pain abdomen (five, 7.7%). The co-occurrence pattern of the symptoms matches that of the entire study population.

**Figure 8:**
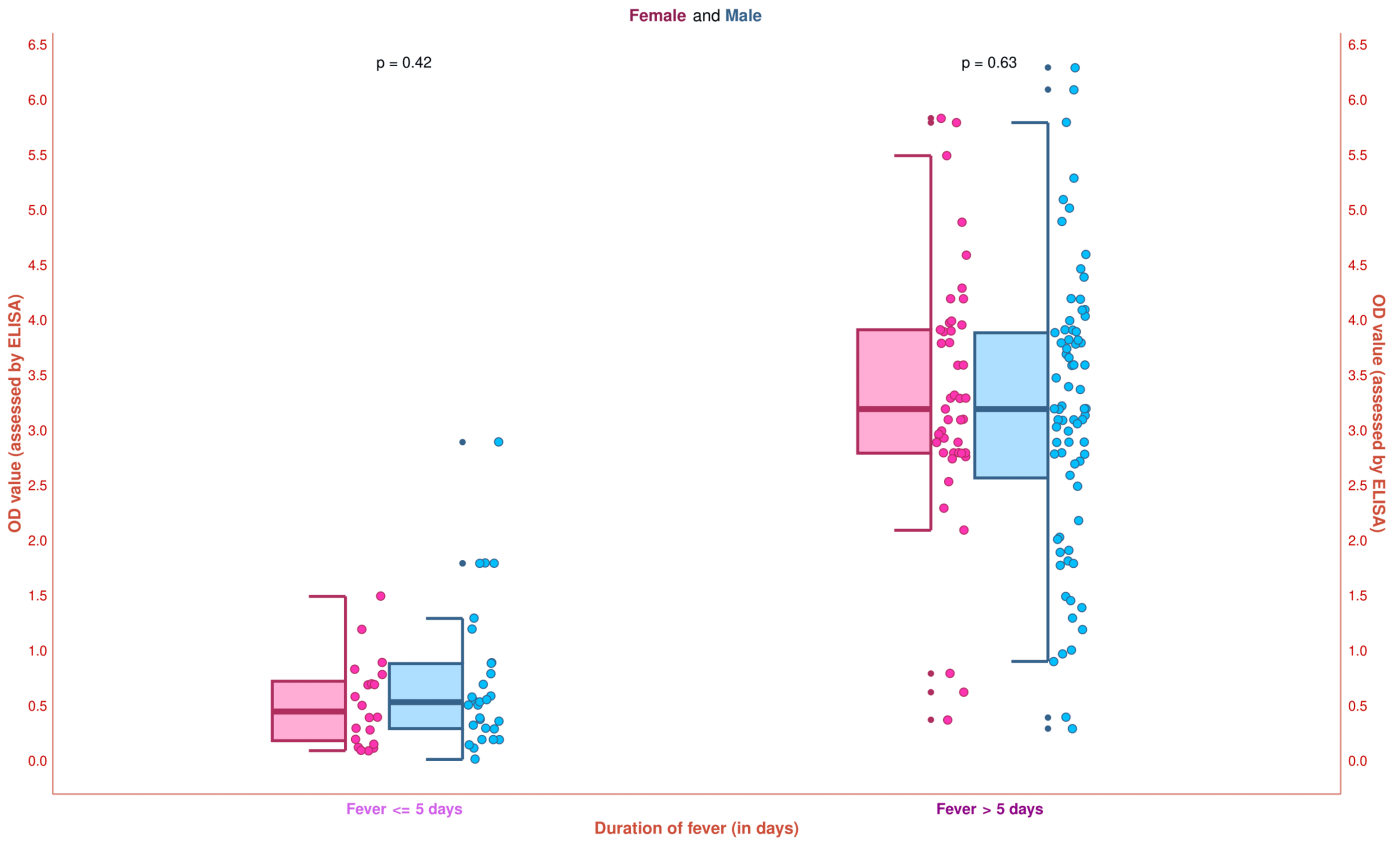
The OD values of the study participants The half-box-whisker and jitter plots demonstrate the OD values of the study participants. The x-axis and y-axis denote the participants’ duration of fever (in days) and OD values (assessed by ELISA), respectively. ELISA: Enzyme-linked immunosorbent assay, OD: Optical density

The half-box-whisker and jitter plots in Figure 8 illustrate the study participants' OD values (assessed by ELISA). The median OD value of the study population was 2.8 (0.8-3.7) (female: 2.8 (0.7-3.6); male: 2.8 (1.0-3.8)). Subgroup analysis was performed based on fever duration. The median OD values for the 20 female and 29 male participants with fever ≤ 5 days were 0.5 (0.2-0.7) and 0.5 (0.3-0.9), respectively (p = 0.42). The median OD values for the 45 female and 76 male participants having fever > 5 days were 3.2 (2.8-3.9) and 3.2 (2.6-3.9), respectively (p = 0.63). These findings indicate that the OD values were increased significantly after five days of febrile illness. The increment in OD values corresponded to the rising IgM antibody titer against *O. tsutsugamushi*. The OD values also advocated that ELISA should be preferred among the scrub typhus patients with fever for > 5 days.

**Figure 9:**
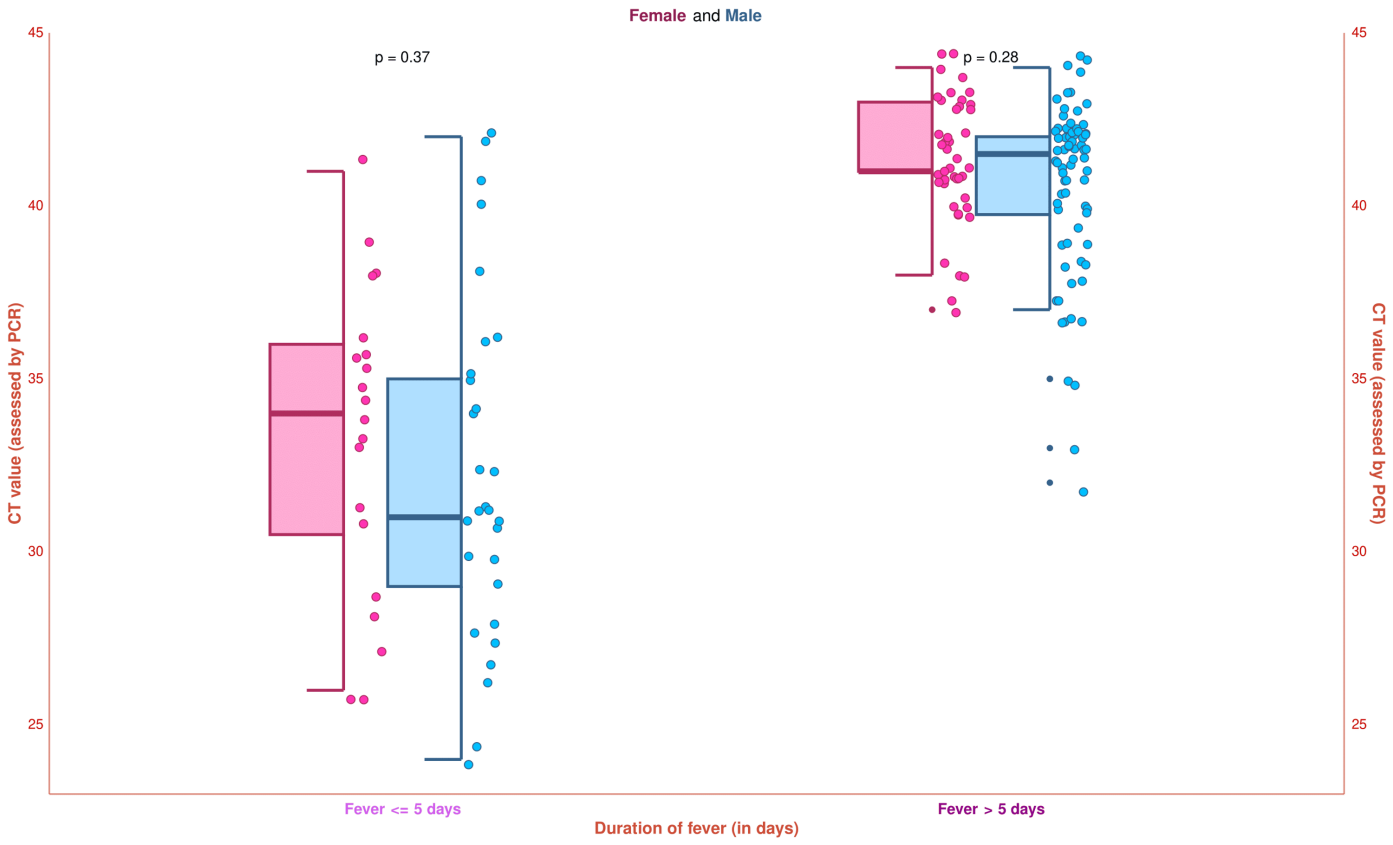
The CT values of the study participants The half-box-whisker and jitter plots demonstrate the CT values of the study participants. The x-axis and y-axis denote the participants’ duration of fever (in days) and CT values (assessed by PCR), respectively. PCR: Polymerase chain reaction, CT: Cycle threshold

The half-box-whisker and jitter plots in Figure 9 illustrate the study participants' CT values (assessed by PCR). The median CT value of the study population was 41.0 (36.0-42.0) (female: 41.0 (37.0-42.0); male: 41.0 (36.0-42.0)). Subgroup analysis was performed based on fever duration. The median CT values for the 20 female and 29 male participants with fever ≤ 5 days were 34.0 (30.5-36.0) and 31.0 (29.0-35.0), respectively (p = 0.37). The median CT values for the 45 female and 76 male participants having fever > 5 days were 41.0 (41.0-43.0) and 41.5 (39.8-42.0), respectively (p = 0.28). These findings indicate that the CT values were increased significantly after five days of febrile illness. The bacterial load decreased after five days as the antibody titer increased. The increasing CT value and reduced bacterial load implied a recent infection by *O. tsutsugamushi*. The CT values also suggested that DNA RT-PCR should be leveraged among the scrub typhus patients with fever for ≤ 5 days.

**Figure 10:**
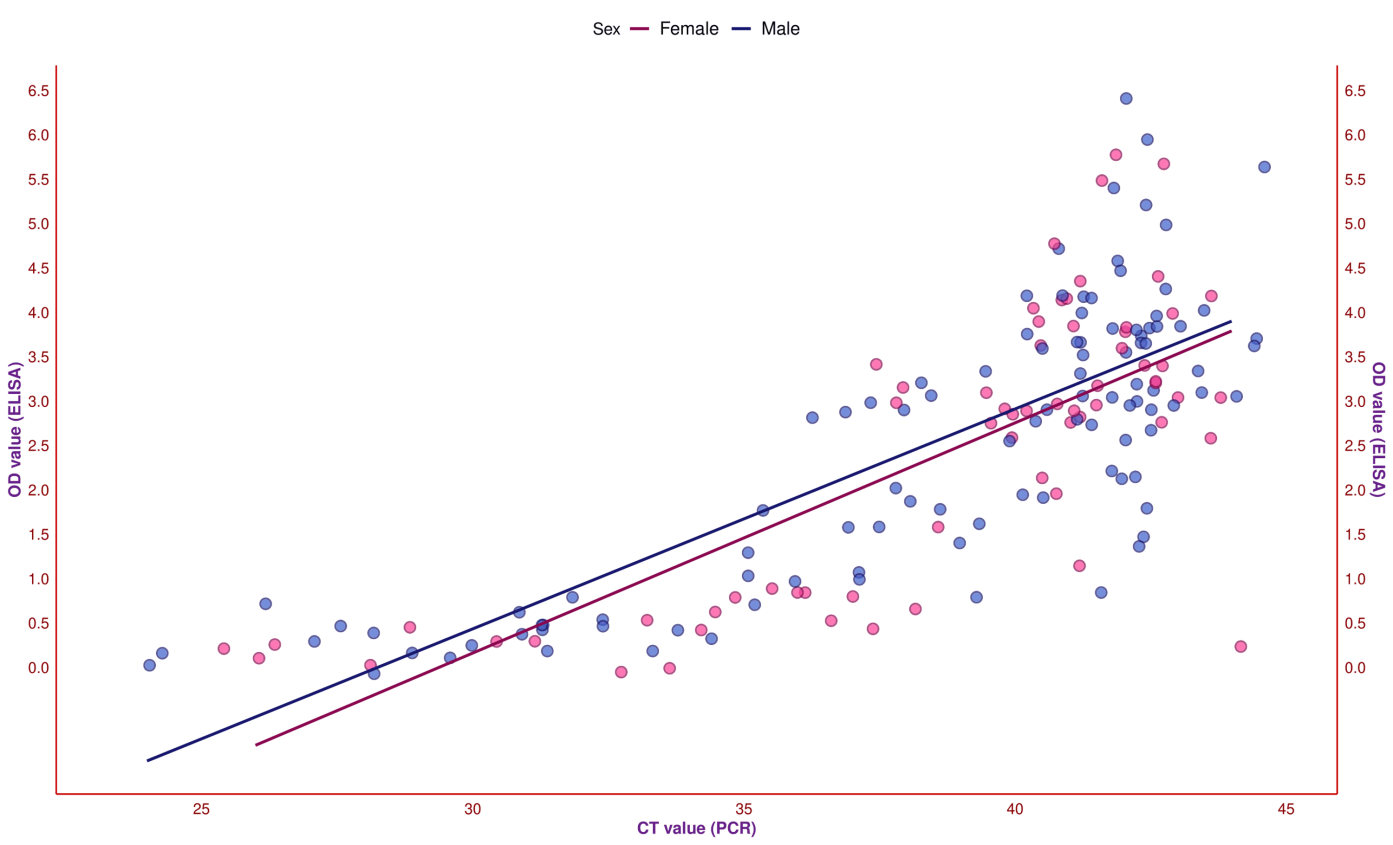
The CT values assessed by PCR The scatter plot demonstrates the correlation between the OD and CT values of the study participants. The x-axis and y-axis denote the participants' CT values (assessed by PCR) and OD values (assessed by ELISA), respectively. The pink and blue dots represent the values for female and male participants, respectively. PCR: Polymerase chain reaction, CT: Cycle threshold, ELISA: Enzyme-linked immunosorbent assay, OD: Optical density

**Figure 11:**
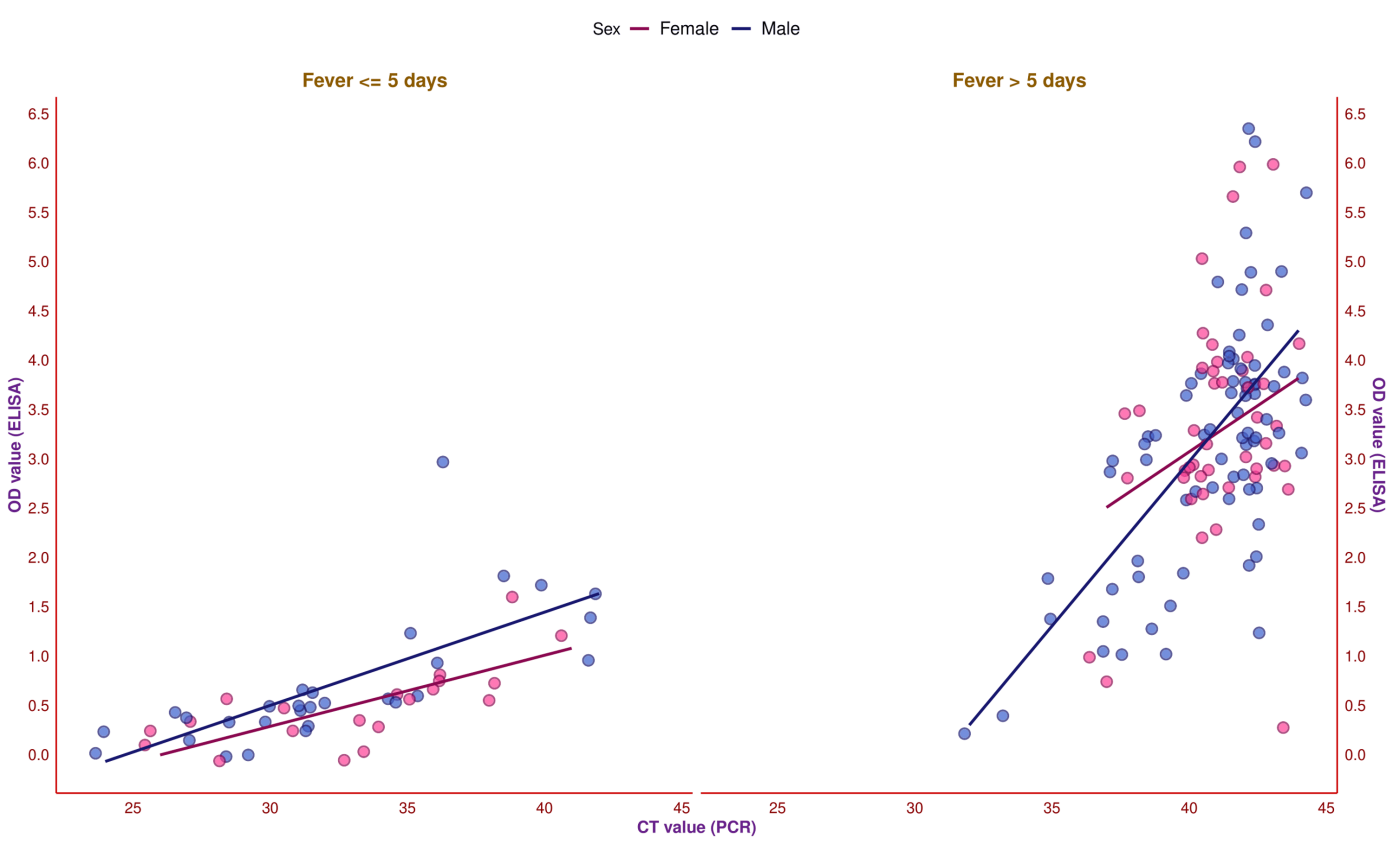
Correlation of OD and CT values based on fever duration The scatter plots display the correlation between the OD and CT values of the study participants. The x-axis and y-axis denote the participants' CT values (assessed by PCR) and OD values (assessed by ELISA), respectively. The pink and blue dots represent the values for female and male participants, respectively. PCR: Polymerase chain reaction, CT: Cycle threshold, ELISA: Enzyme-linked immunosorbent assay, OD: Optical density

The scatter plot in Figure 10 showcases the correlation between the OD and CT values of the study participants. These two values had a positive correlation (r = 0.78, 95% CI = 0.71-0.83, p < 0.001). For the 65 female participants, the OD and CT values were positively associated (r = 0.75, 95% CI = 0.62-0.84, p < 0.001). For the 105 male participants, the OD and CT values were also positively associated (r = 0.79, 95% CI = 0.71-0.85, p < 0.001). To gauge the correlation based on antibody titer, we analyzed the correlation between OD and CT values per the fever duration (Figure 11). The OD and CT values were positively associated with the 20 female subjects with fever ≤ 5 days (r = 0.82, 95% CI = 0.59-0.92, p < 0.001). There was a positive correlation between the OD and CT values for the 29 male subjects with fever ≤ 5 days (r = 0.73, 95% CI = 0.49-0.86, p < 0.001). The OD and CT values were positively associated with the 45 female subjects with fever > 5 days (r = 0.30, 95% CI = 0.01-0.54, p = 0.047). There was also a positive correlation between the 76 male subjects with fever > 5 days (r = 0.66, 95% CI = 0.51-0.77, p < 0.001) between the OD and CT values. The correlation coefficients among the participants with a lesser duration of febrile illness were higher when juxtaposed with those with chronic fever.

## Discussion

In this retrospective study, 170 patients with scrub typhus were analyzed. The study population's median age was 44.5 (24.0-61.8) years. Most participants (98, 57.6%) belonged to a lower socioeconomic class. The entire study population had febrile illness during hospital admission. Gautam et al. [18] also found that 100% of participants had fever. Cough (139, 81.8%), lymphadenopathy (29, 17.1%), rash (26, 15.3%), abdominal pain (18, 10.6%), eschar (6, 3.5%), and seizure (2, 1.2%) were the other symptoms among our study subjects. The maximum co-occurrence was observed between cough and fever. Febrile illness was also commonly noticed along with symptoms like lymphadenopathy, rash, and pain in the abdomen. It held good for both females and males. The study population's median OD and CT values were 2.8 (0.8-3.7) and 41.0 (36.0-42.0), respectively. The OD and CT values had a positive correlation (r = 0.78, 95% CI = 0.71-0.83, p < 0.001). Anitharaj et al. [20] and Gatika et al. [21] employed both IgM ELISA and PCR in their study. Nonetheless, they did not present the OD and CT values of the participants.

Scrub typhus is a multi-system infection imparting significant mortality and morbidity. It can even lead to myocarditis, acute kidney damage, meningoencephalitis, acute respiratory distress syndrome (ARDS), and disseminated intravascular coagulation (DIC) [22]. The Indian state of Odisha has lately reported an increasing trend of scrub typhus cases [3]. Symptom diversity, shortage of resources and infrastructure, and lack of cost-effective and quick diagnostic methods have challenged the scrub typhus diagnosis [3,8,9]. We selected IgM ELISA and DNA RT-PCR as diagnostic tools to gauge the study subjects' IgM antibody titer and bacterial load. Our observations unveiled that ELISA could be opted for diagnosis for scrub typhus patients with > 5 days of fever, as antibody rise mandates three to five days of infection. We also noticed that RT-PCR could serve as a diagnostic tool for scrub typhus in the earlier days of presentation owing to the growing bacterial load. Our observations concorded with the studies by Saraswati et al. [10] and Konyak et al. [14]. 

As fever and cough were the two most common symptoms in our study population, they could be presumed to have higher co-occurrences than other symptoms. Rash and pain in the abdomen also demonstrated co-occurrence despite their lower incidences among the males. However, we reported lower incidences of eschar in the scrub typhus patients. In an Indian study by Sivarajan et al. [23], it was revealed that the incidence of eschar was outnumbered by fever, lymphadenopathy, and rash.

The OD and CT values in the study population were increased significantly after five days of febrile illness. The increment in OD values corresponds to the rising IgM antibody titer against O. tsutsugamushi. The bacterial load decreased after five days as the antibody titer increased. The increasing CT values and reduced bacterial loads implied a recent infection by O. tsutsugamushi. Compared to patients with chronic fever, those with shorter durations of febrile illness had greater correlation coefficients between OD and CT values. Scrub typhus cases can only be confirmed by reliable diagnostic tests like ELISA and PCR and greater clinical suspicion. Underneath the "One Health" umbrella, all relevant organizations, especially the grassroots stakeholders, could team up to investigate the scrub typhus cases [24].

This study's main strength was the presentation of symptom co-occurrence through heatmap diagrams. Our study had some limitations as well. First, the retrospective study design restricted the sample size. Second, we did not correlate the duration of symptoms, hospital stay, and antibiotic administration. Third, we did not assess the impact of antibiotics on the OD and CT values. Fourth, we also did not analyze any sociodemographic traits with these values.

## Conclusions

The study subjects had rash, eschar, fever, cough, lymphadenopathy, and abdominal pain. According to our research, ELISA and DNA RT-PCR could serve as diagnostic tools for scrub typhus per the fever duration. A positive association was discovered between the two diagnostic techniques. Human physicians, veterinarians, community health specialists, ecologists, and environmentalists must address the new concerns posed by scrub typhus. We suggest conducting more prospective research with bigger sample sizes to assess the correlation between DNA RT-PCR and ELISA findings.
